# Artificial intelligence-based remote monitoring for chronic heart failure: design and rationale of the SMART-CARE study

**DOI:** 10.3389/fdgth.2025.1719562

**Published:** 2025-12-10

**Authors:** Michele Ciccarelli, Alessia Bramanti, Albino Carrizzo, Marina Garofano, Valeria Visco, Carmine Izzo, Maria Rosaria Rusciano, Gennaro Galasso, Francesco Loria, Giorgia Bruno, Carmine Vecchione

**Affiliations:** 1 Department of Medicine, Surgery and Dentistry, University of Salerno, Baronissi, Italy; 2 Vascular Pathophysiology Unit, IRCCS Neuromed, Isernia, Italy; 3 Department of Cardiac Rehabilitation, University Hospital “San Giovanni di Dio e Ruggi d'Aragona”, Salerno, Italy

**Keywords:** chronic heart failure, remote monitoring, artificial intelligence, wearable devices, digital health

## Abstract

**Introduction:**

Chronic heart failure (CHF) is associated with frequent hospitalizations, poor quality of life, and high healthcare costs. Despite therapeutic progress, early recognition of clinical deterioration remains difficult. The SMART-CARE study investigates whether artificial intelligence (AI)-enabled remote monitoring using CE-certified wearable devices can reduce hospital admissions and improve patient outcomes in CHF.

**Methods:**

SMART-CARE is a prospective, multicenter, observational cohort study enrolling 300 adult patients with CHF (HFrEF, HFmrEF, or HFpEF) across three Italian tertiary centers. Participants are assigned to an intervention group, using wrist-worn, chest-worn, and multiparametric CE-certified wearable devices for six months, or to a control group receiving standard CHF care. Physiological data (e.g., SpO₂, HRV, respiratory rate, skin temperature, sleep metrics) are continuously collected and analyzed in real time through AI algorithms to generate alerts for early clinical intervention. The primary endpoint is a ≥20% reduction in hospital admissions over six months. Secondary outcomes include changes in quality of life (Kansas City Cardiomyopathy Questionnaire), biomarkers (BNP, NT-proBNP, renal function, electrolytes), echocardiographic indices (LVEF, LV volumes), and safety events.

**Results:**

We hypothesize that AI-driven remote monitoring will significantly reduce hospitalizations, improve quality of life, and favorably impact biochemical and echocardiographic parameters compared to standard care.

**Conclusion:**

SMART-CARE is designed to evaluate the clinical utility of multimodal wearable devices integrated with AI algorithms in CHF management. If successful, this approach may transform traditional care by enabling earlier detection of decompensation, optimizing resource utilization, and supporting the scalability of remote monitoring in chronic disease management.

**Clinical Trial Registration:**

ClinicalTrials.gov, identifier NCT06909682.

## 1 Introduction

The incidence of chronic diseases is progressively increasing, along with the associated care burden for families, general practitioners, and the National Health System (1–3). Patients affected by chronic conditions are complex and require strong interaction between primary care and specialized centers to ensure proper management of all phases of the disease, such as diagnosis, clinical exacerbation handling, and the definition of an individualized therapeutic program (4). Specifically, chronic heart failure (CHF) is a complex syndrome involving multiple regulatory systems and is associated with high rates of rehospitalization and mortality (5). The complexity of this condition often necessitates frequent access to hospital facilities for multidisciplinary evaluations and diagnostic exams, thereby increasing both direct and indirect costs (lost workdays, travel, inappropriate hospitalizations) (6, 7). For patients with multiple chronic conditions, whose continuous therapies and follow-up visits aim to extend the number of years lived without severe disabilities, it is crucial to create a “territorial network” that facilitates dialogue between local health services and secondary/tertiary care centers (8). This would promote resources optimization and reduce hospital admissions or services. Furthermore, appropriate management during the intervals between outpatient visits is essential. In recent years, there has been a growing adoption of telemedicine solutions, which, through information and communication technologies (ICT), enable the delivery of healthcare services outside traditional healthcare settings, even when the healthcare provider and patient are in different locations (9, 10). Telemedicine is a valuable tool that supports the continuity of care between hospitals and communities, improves access to health services, and optimizes chronic disease management (11, 12). It also helps enhance home care and reduce unnecessary travel, especially for patients living in remote areas (13). ICT is used to facilitate the exchange of information between care partners and to promote patient empowerment by providing devices for self-monitoring of biomedical parameters, which can be remotely monitored by healthcare professionals to tailor the treatment plan (14). CHF, in particular, is characterized by alternating periods of stability and clinical worsening, but over the last three decades, management of this condition has significantly advanced, with pharmacological and device-based therapies (15, 16). Among these, implantable defibrillators, alone or combined with cardiac resynchronization therapy, are equipped with algorithms that monitor signs of congestion through thoracic impedance. Despite their effectiveness, these devices are invasive and require surgical implantation (17, 18). Given the high burden and complexity of CHF, there is an increasing need for complementary data beyond clinical history and physical examination, including telemonitoring and biochemical information (19). In recent years, the most innovative scenario has been represented by disease management supported by wearable and non-invasive medical devices capable of continuously collecting physiological parameters useful for remote monitoring. These technologies are available in different formats—wrist-worn devices, chest sensors, and non-invasive multiparametric monitors—each with specific advantages. Among them, the Empatica EmbracePlus (Empatica Inc.) is a CE-certified wristband capable of recording multiple signals such as blood oxygen saturation (SpO₂), heart rate (HR) and variability (HRV), electrodermal activity (EDA), sleep quality (actigraphy), respiratory rate (RR), temperature, and fatigue detection (20–22). The Movesense MD sensor (Suunto/Kaasa Health) is a CE-marked chest-worn device validated for ECG and motion tracking (23), while the Polar H10 chest strap (Polar Electro), although primarily designed for sports and fitness, provides accurate HR and HRV measurements that can be integrated into telemonitoring programs (24, 25). In addition, a non-invasive multiparametric device allows real-time monitoring of several metabolic and hemodynamic variables (e.g., glucose, hemoglobin, blood pressure, and oxygen saturation), expanding the range of information available for clinical decision-making (26). When integrated with telemedicine platforms, electronic health records, and advanced analytics, these devices enable continuous monitoring, early detection of decompensation, and timely therapeutic adjustments (e.g., diuretic dosage). This comprehensive and patient-centered approach has the potential to improve self-management, optimize healthcare resource utilization, and reduce CHF exacerbations and hospitalizations. However, despite technological progress, most previous telemonitoring studies in CHF have relied on single-parameter measurements, intermittent data collection, or rule-based alert systems (27–29), which limit their ability to detect subtle and individualized early signs of clinical deterioration. Moreover, only a few studies have integrated continuous multimodal wearable signals, and the application of advanced artificial intelligence to real-time physiologic data remains limited (30, 31). SMART-CARE aims to address this knowledge gap by combining high-resolution, multi-sensor wearable data with AI-driven predictive analytics capable of identifying trends, detecting early physiological deviations, and generating personalized alerts. This approach is designed to overcome the limitations of earlier telemonitoring interventions and support a more proactive and individualized model of CHF management.

This study aims to collect foundational data for the development of the “SMART-CARE” project. The project's final goal is to identify relationships and/or mathematical formulas (multimarker scores) among clinical, biochemical, instrumental, and wearable-monitored parameters that can predict the course and prognosis of patients with CHF. Within this study protocol, part of the broader SMART-CARE project, the objective is to assess whether remote monitoring using a wearable, mini-invasive device can reduce hospitalizations in patients with CHF.

## 2 Methods

### 2.1 Study design and population

**Figure 1:**
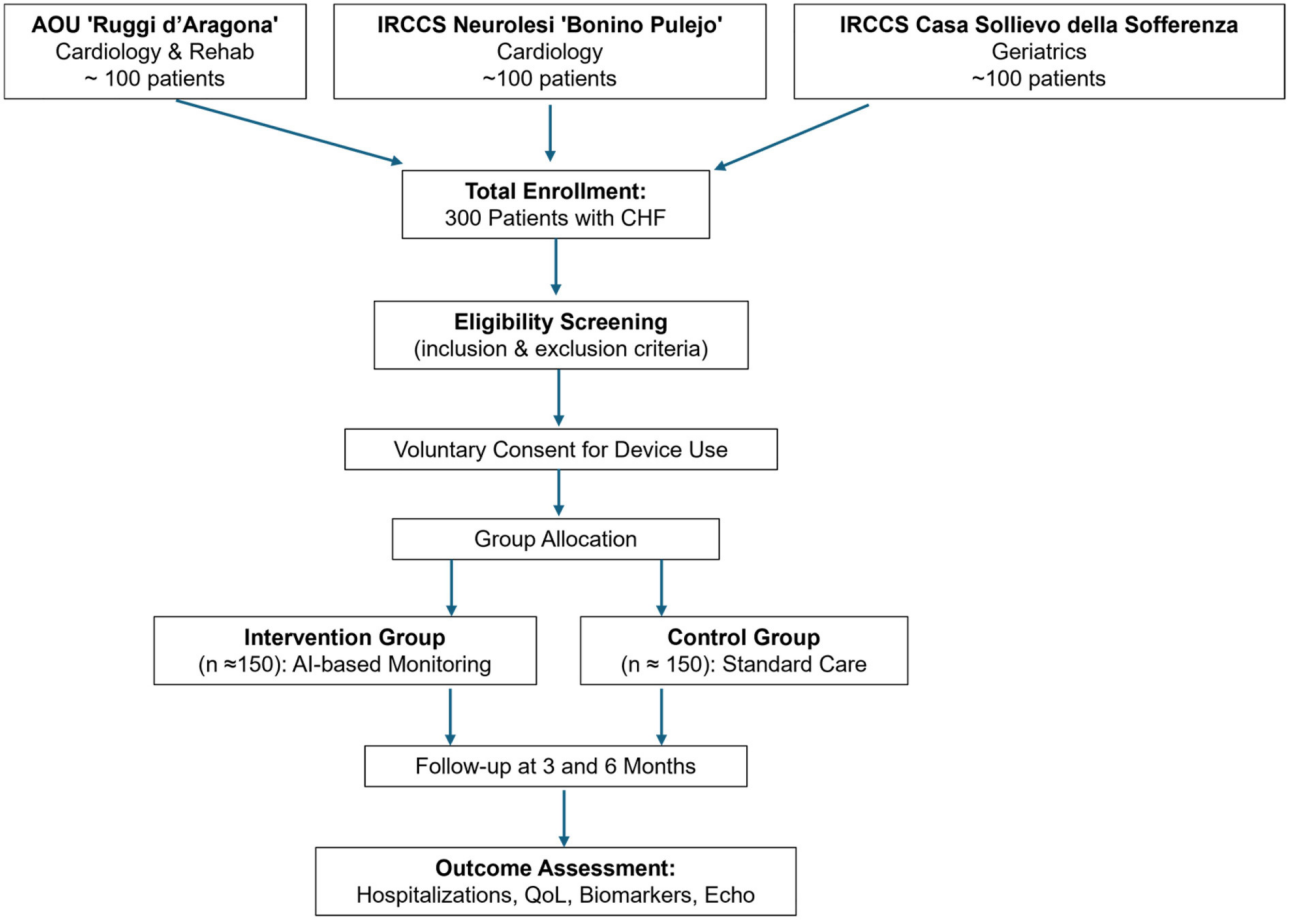
Flow diagram of the study design.

The SMART-CARE study is a multicenter, prospective, observational cohort trial, designed to evaluate the clinical utility of artificial intelligence (AI)-enabled remote monitoring in patients with CHF. A prospective, non-randomized observational design was chosen for both ethical and pragmatic reasons. Allocation to the intervention group required patients' explicit willingness to use the wearable device and to undergo continuous monitoring, making randomization unfeasible. To minimize potential selection bias, statistical adjustment methods (multivariable regression, propensity score matching, and stratified analyses by age, sex, New York Heart Association class - NYHA, Left Ventricular Ejection Fraction - LVEF phenotype, and comorbidities) will be applied. The trial will be conducted across three tertiary care centers in Italy: AOU “San Giovanni di Dio Ruggi d'Aragona”, IRCCS Neurolesi “Bonino Pulejo”, and IRCCS Fondazione Casa Sollievo della Sofferenza. The study will enroll 300 adult patients (≥18 years) with a confirmed diagnosis of CHF, including Heart Failure with reduced Ejection Fraction (HFrEF), Heart Failure with mildly reduced Ejection Fraction (HFmrEF), or Heart Failure with preserved Ejection Fraction (HFpEF) for at least six months, who are clinically stable and on optimized medical therapy for at least one month prior to enrolment (Figure 1).

Eligible patients will be stratified into two parallel cohorts based on voluntary consent to participate in the remote monitoring intervention. Patients in the intervention group will wear the EmbracePlus (Empatica Inc.) mini-invasive device continuously for six months (20, 21). This CE-certified wearable device captures a comprehensive set of physiological parameters, including SpO₂, HRV, EDA, respiratory rate, peripheral skin temperature, and sleep quality, which are transmitted in real time to the SMART-CARE digital platform (20–22). The collected data will be processed through AI algorithms capable of identifying abnormal trends and generating alerts to prompt timely clinical action (32–34); moreover, these data will serve a broader purpose within the SMART-CARE project: to support the development of a novel diagnostic and prognostic algorithm. By integrating biometric signals with clinical, instrumental, and biochemical information, the SMART-CARE platform will apply advanced machine learning techniques to generate predictive models aimed at identifying the clinical trajectory of CHF patients and optimizing personalized care strategies.

Patients in the control group will undergo standard chronic heart failure (CHF) follow-up as per clinical guidelines, including in-person visits every three months, electrocardiography (ECG), echocardiography, and laboratory evaluation [e.g., B-type Natriuretic Peptide (BNP), N-terminal pro-B-type Natriuretic Peptide (NT-proBNP), renal and hepatic function, serum electrolytes]. All patients, regardless of group assignment, will be evaluated at baseline, 3 months, and 6 months.

Patients with NYHA class IV symptoms, anticipated heart transplantation or ventricular assist device implantation within 6 months, severe renal impairment (estimated Glomerular Filtration Rate - eGFR <30 mL/min/1.73 m^2^), terminal illness, or inability to comply with study procedures will be excluded (Table 1).

**Table 1:** Inclusion and exclusion criteria for the SMART-CARE study.

Inclusion criteria	Exclusion criteria
Age ≥ 18 years (male or female)	NYHA class IV symptoms
Confirmed diagnosis of CHF ≥ 6 months prior to screening	Planned heart transplant or VAD implantation within 6 months
NYHA functional class I, II, or III	Severe renal impairment (eGFR < 30 mL/min/1.73 m^2^) or dialysis dependency
Optimized heart failure therapy for at least 1 month prior to enrolment	Terminal illness with limited life expectancy (e.g., metastatic cancer, end-stage lung disease)
Any LVEF phenotype (HFrEF, HFmrEF, HFpEF)	Pregnancy
≥1 hospitalization or outpatient visit with IV therapy for CHF in last 12 months	Dermatological conditions or allergies preventing prolonged use of the wearable device
Ability to provide informed consent or availability of a legally authorized representative	Cognitive or psychiatric disorders impairing adherence to study procedures

CHF, chronic heart failure; eGFR, estimated Glomerular Filtration Rate; HFmrEF, Heart Failure with mildly reduced Ejection Fraction; HFpEF: Heart Failure with preserved Ejection Fraction; HFrEF, Heart Failure with reduced Ejection Fraction; IV, intravenous; LVEF, left ventricular ejection fraction; NYHA, New York Heart Association; VAD, ventricular assist device.

All clinical, instrumental, and biochemical data will be recorded in anonymized case report forms and stored in a secure digital database. Biospecimens (blood and urine) will be collected and retained for secondary biomarker analysis. The study protocol adheres to the Declaration of Helsinki and Good Clinical Practice guidelines, and it was approved by the regional ethics committee (Comitato Etico Campania 2; Approval No. 185867). The trial is registered at ClinicalTrials.gov with the identifier NCT06909682.

### 2.2 Groups and interventions

Participants will be allocated to one of two non-randomized, parallel groups based on their acceptance to use the wearable device:

- Intervention Group – AI-Based Remote Monitoring: Patients in this group will wear wearable devices continuously over a six-month period. The wearable device collects multimodal biometric data, including SpO₂, HRV, EDA, respiratory rate, skin temperature, pulse rate, and sleep metrics (actigraphy). These data are automatically transmitted to the SMART-CARE platform, where advanced AI algorithms analyse trends and detect early signs of clinical deterioration. In case of abnormal findings, the system generates alerts for the clinical team, which may initiate teleconsultations, therapy adjustments, or in-person evaluations to prevent acute decompensation.
- Control Group – Standard Clinical Follow-Up: Participants in the control group will receive standard CHF management in accordance with current European Society of Cardiology (ESC) guidelines. This includes outpatient visits every three months, ECG and echocardiographic assessments, and laboratory evaluations. No remote monitoring will be applied in this group. Clinical decisions, including medication adjustments, will be based on periodic clinical evaluations and patient-reported symptoms.

### 2.3 Technological solution used for the AI-based remote monitoring group

The architecture is compliant with the General Data Protection Regulation (GDPR) and implements international interoperability standards (HL7/FHIR), thereby allowing integration with local electronic health records (EHRs) and hospital information systems. It is organized into layered components to guarantee scalability and flexibility:

1. An access layer providing secure authentication, authorization, and full audit trails;
2. An application layer incorporating a patient dashboard for real-time visualization of biometric trends, a workflow engine for automated monitoring and alert-response processes, videoconferencing and chat modules to support synchronous and asynchronous communication, and questionnaire/PRO management tools;
3. A service layer including a message broker for asynchronous communication, a complex event processing engine to transform raw IoT streams into clinically relevant events, and machine learning services for predictive analytics;
4. A data layer responsible for the secure storage of structured, semi-structured, and unstructured data, ensuring persistence of time series, medical imaging, and laboratory information in compliance with international standards.

On the patient side, the monitoring system relies on an integrated set of wearable and IoT devices. These include the EmbracePlus wearable (Empatica Inc.), selected for its CE-certification, multi-sensor capability, and validation in continuous physiological monitoring, as well as additional complementary devices such as blood pressure monitors, weight scales, glucometers, pulse oximeters, wearable movement sensors, and non-invasive hemodynamic monitoring systems. Together, these tools enable a comprehensive assessment of physiological parameters and patient status.

Data collected by the devices are transmitted via Bluetooth and encrypted cloud channels to the SMART-CARE platform, where they are processed by AI algorithms designed to detect abnormal trends and generate alerts for timely clinical action.

The artificial intelligence component of the SMART-CARE study will rely on an explainable, non-commercial predictive model based on Genetic Programming (GP), as recently described by Visco et al. (2024) (35). This algorithm employs a supervised learning framework in which clinical, biochemical, instrumental, and wearable-derived parameters are integrated through symbolic regression to generate transparent mathematical expressions. GP autonomously selects the most informative features and constructs human-interpretable predictive formulas, enabling clear visualization of variable contributions and overcoming the limitations of black-box AI models. This approach is particularly suitable for multimodal datasets collected in SMART-CARE, allowing real-time identification of early physiological deviations and supporting clinical explainability and bias assessment. The GP model will be adapted and retrained on the SMART-CARE cohort to evaluate its ability to predict early signs of worsening heart failure when combined with continuous wearable-derived physiological signals.

**Figure 2:**
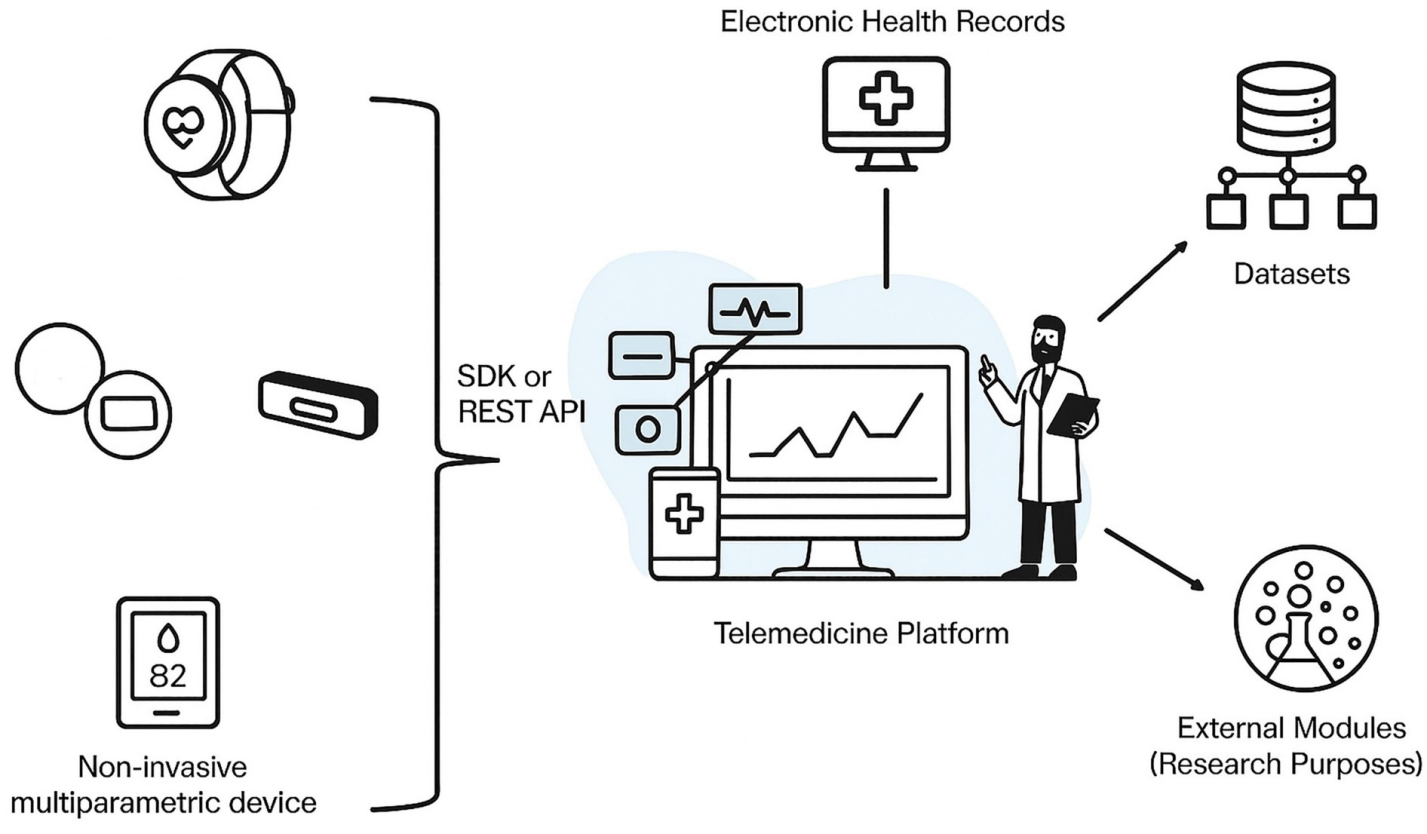
Overview of the SMART-CARE telemedicine platform architecture. Wearable and multiparametric devices (EmbracePlus, Movesense/Polar H10, additional IoT sensors) transmit data via SDK/API REST to the telemedicine platform, which integrates with electronic health records, generates research datasets, and allows interoperability with external modules for advanced analytics.

This integrated technological ecosystem ensures interoperability, scalability, and real-time responsiveness, enabling a truly AI-driven approach to remote monitoring and fostering the incorporation of digital health tools into routine clinical workflows (Figure 2).

This integrated technological ecosystem ensures interoperability, scalability, and real-time responsiveness, enabling a truly AI-driven approach to remote monitoring and fostering the incorporation of digital health tools into routine clinical workflows.

### 2.4 Outcomes

Both groups will undergo assessments at baseline, 3 months, and 6 months, including evaluations of functional status, echocardiographic parameters, biochemical markers (e.g., BNP, NT-proBNP, renal function), and quality of life assessed using the Kansas City Cardiomyopathy Questionnaire (KCCQ) (36–39) (Table 2).

**Table 2:** SMART-CARE outcome measures by timepoint.

Outcome type	Outcome measure	Domain	Baseline	3 months	6 months
Primary	Change in Hospital Admissions	Clinical Events			✔
Secondary	Change in Quality of Life (KCCQ Score)	Quality of Life	✔	✔	✔
Secondary	Adverse Effects of CHF Therapy	Safety			✔
Secondary	Change in BNP Levels	Biochemical	✔	✔	✔
Secondary	Change in NT-proBNP Levels	Biochemical	✔	✔	✔
Secondary	Change in Serum Creatinine	Biochemical	✔	✔	✔
Secondary	Change in eGFR	Biochemical	✔	✔	✔
Secondary	Change in Serum Sodium	Biochemical	✔	✔	✔
Secondary	Change in Serum Potassium	Biochemical	✔	✔	✔
Secondary	Change in Serum Chloride	Biochemical	✔	✔	✔
Secondary	Change in HRV	Functional (ECG)	✔	✔	✔
Secondary	Change in Respiratory Rate	Functional (ECG)	✔	✔	✔
Secondary	Change in LVEF	Echocardiographic	✔	✔	✔
Secondary	Change in E/A Ratio	Echocardiographic	✔	✔	✔
Secondary	Change in LV End-Diastolic Volume	Echocardiographic	✔	✔	✔
Secondary	Change in LV End-Systolic Volume	Echocardiographic	✔	✔	✔

CHF, chronic heart failure; KCCQ, Kansas City Cardiomyopathy Questionnaire; BNP, B-type Natriuretic Peptide; NT-proBNP, N-terminal pro-B-type Natriuretic Peptide; eGFR, estimated Glomerular Filtration Rate; HRV, Heart Rate Variability; ECG, Electrocardiogram; LVEF, Left Ventricular Ejection Fraction; E/A Ratio, Early to Late Ventricular Filling Velocity Ratio; LV, left ventricle.

The primary outcome of the SMART-CARE study is the relative reduction (≥20%) in hospital admissions, including emergency department visits and unplanned hospitalizations, over a six-month follow-up period in the intervention group compared to standard care (40). Hospitalization data will be collected prospectively and analysed for frequency, duration, and cause.

Secondary outcomes include:

- Quality of Life (QoL): Measured using KCCQ at baseline, 3 months, and 6 months.
- Biochemical parameters: BNP, NT-proBNP, serum creatinine, eGFR, and serum electrolyte levels (sodium, potassium, chloride) at 3 and 6 months.
- Functional parameters: Variations in HRV and RR derived from wearable sensor data.
- Echocardiographic measures: LVEF, Early to Late Ventricular Filling Velocity Ratio (E/A ratio), and left ventricular end-diastolic and end-systolic volumes, measured at 3 and 6 months.
- Adverse events: Incidence of therapy-related complications such as hypotension and bradyarrhythmias.

These outcomes will provide a comprehensive evaluation of the impact of AI-driven remote monitoring clinical status, functional performance, biomarker trends, and patient-reported outcomes in individuals with CHF. The measurements collected by the minimally invasive devices will be uploaded to the “Smartcare” platform and analyzed using machine learning (ML) techniques to develop multiparametric scores capable of identifying the specific trajectory of patients affected by HF.

### 2.5 Safety management and contingency planning

An additional strength of the SMART-CARE design lies in the structured approach to emergency management. The platform is not limited to early detection but also integrates a predefined pathway to ensure patient safety in the event of acute clinical deterioration. Alerts generated by abnormal biometric trends prompt immediate evaluation by the clinical team, who can escalate the response according to severity—ranging from direct patient contact or urgent teleconsultation to expedited in-person assessment or activation of local emergency medical services (EMS, 112/118 in Italy). This structured hospital–community network ensures rapid intervention and continuity of care, thereby minimizing the risk of adverse outcomes and reinforcing the overall safety and feasibility of AI-driven remote monitoring in real-world settings.

**Figure 3:**
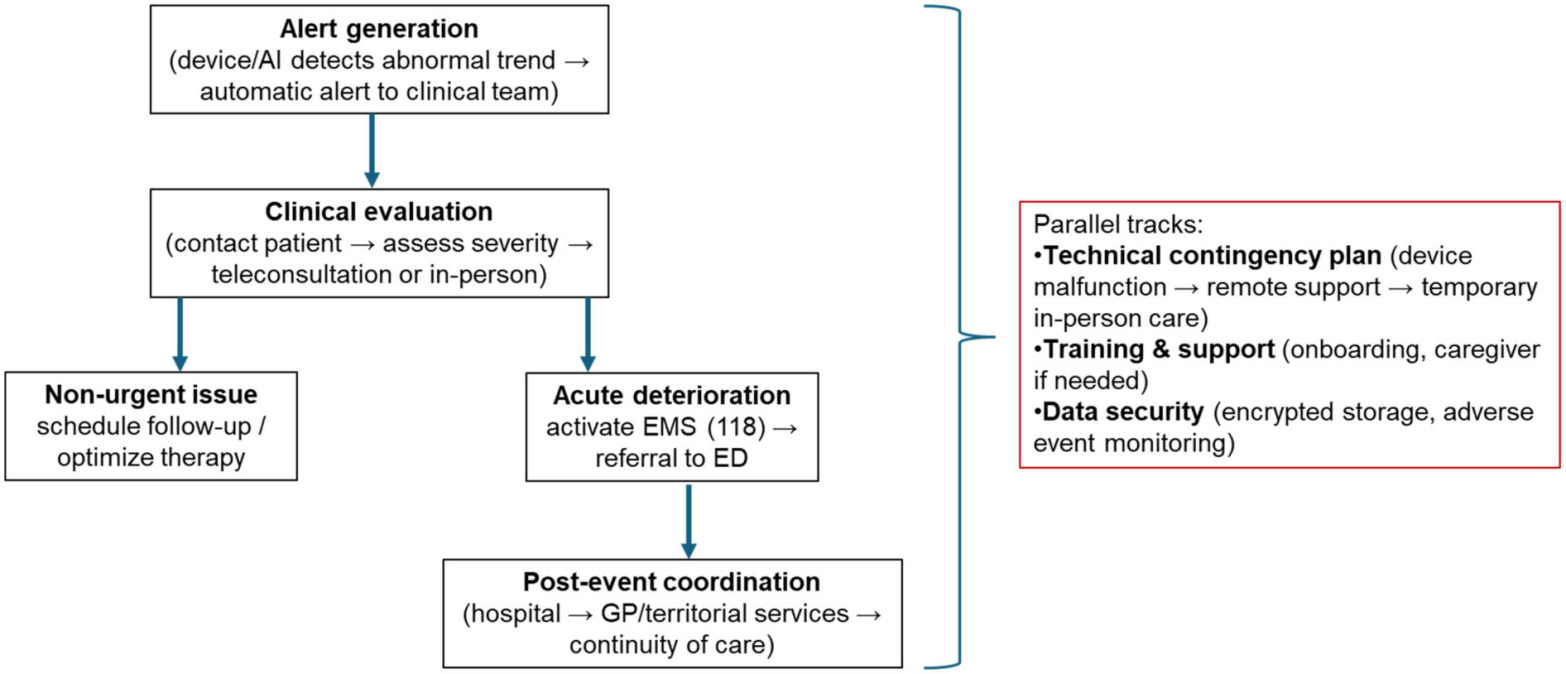
SMART-CARE emergency management pathway. The right panel summarizes cross-cutting safety measures (technical contingency, training and support, data security) that are active across all phases of the clinical workflow.

In addition, a technical contingency plan is in place: in case of device malfunction, connectivity issues, or platform failure, patients are promptly supported by remote technical assistance, with the possibility of temporary transition to standard in-person care to guarantee uninterrupted clinical follow-up. All participants undergo structured onboarding, including device training and safety instructions, and those with limited digital literacy are required to have caregiver support; continuous technical and clinical assistance is available throughout the study period. Furthermore, all clinical and biometric data are transmitted and stored in secure, encrypted repositories, ensuring data protection, traceability, and real-time safety oversight. This integrated safety and contingency framework is designed to minimize clinical risks, enable rapid response to emergencies, and maintain high standards of care within a remote monitoring setting (Figure 3).

### 2.6 Statistical analysis

#### 2.6.1 Primary endpoint analysis

To assess the primary endpoint of the study, Kaplan–Meier survival curves will be constructed to compare the time to first hospitalization between groups. Group differences will be evaluated using the log-rank test. Subsequently, a Cox proportional hazards regression analysis model will be performed to examine the association between the variable “Device Use (Yes vs. No)” and the reduction in hospital admissions. The model will be adjusted for all statistically significant covariates identified through ANOVA between the two groups. To mitigate selection bias resulting from the non-randomized design, propensity score methods will be applied. The propensity score will be estimated using baseline variables including age, sex, LVEF phenotype, NYHA class, major comorbidities (hypertension, atrial fibrillation, diabetes mellitus, COPD/OSA, obesity), prior HF hospitalizations, baseline biomarkers (BNP/NT-proBNP, renal function, electrolytes), and key echocardiographic indices (LVEF, LV volumes). A 1:1 nearest-neighbour matching approach without replacement will be used. Covariate balance between groups will be evaluated using standardized mean differences (SMD), with values <0.10 indicating adequate balance. Results will be expressed as hazard ratios (HR) with corresponding 95% confidence intervals (CI).

#### 2.6.2 Secondary endpoint analysis

Continuous variables will be presented as mean ± standard deviation (SD) for normally distributed data and as median and interquartile range (IQR) for non-normally distributed data. Categorical variables will be expressed as frequencies and percentages. The normality of distribution will be tested using the Shapiro–Wilk test. For normally distributed continuous variables, comparisons will be made using the paired t-test or one-way repeated measures ANOVA, depending on the situation. For non-normally distributed data, the Wilcoxon Signed-Rank test will be applied. Categorical variables will be compared using the Pearson chi-square test. Given the intrinsic differences in echocardiographic and clinical trajectories between HFrEF and HFpEF patients, all secondary endpoints (including echocardiographic parameters, biomarkers, and functional indices) will also undergo stratified analyses by LVEF phenotype. This approach is intended to avoid the dilution of clinically meaningful changes in subgroups with limited baseline variability, particularly among HFpEF patients. Subgroup analyses based on LVEF phenotype (HFrEF, HFmrEF, HFpEF) will be performed as exploratory analyses. The study is not powered to detect statistically significant differences between these subgroups; therefore, these analyses are intended to be descriptive and hypothesis-generating. To mitigate potential confounding when comparing phenotypes, LVEF category will be included as a covariate in multivariable models, and propensity score techniques will be applied when appropriate. Because echocardiographic parameters show markedly different baseline ranges and variability in HFrEF and HFpEF, echocardiographic secondary outcomes will also be interpreted within each LVEF phenotype. This approach is intended to avoid the dilution of true within-group changes in HFpEF patients, in whom structural parameters often fall within or near the normal range. A two-sided *p*-value < 0.05 will be considered statistically significant for all final analyses.

#### 2.6.3 Sample size calculation

Based on the primary objective of the study, a sample size calculation was performed using G*Power software. The estimation assumes a 20% relative reduction in the rate of hospital admissions (including emergency department visits and hospitalizations) in the intervention group compared to the control group. Using a two-sided alpha of 0.05 and a statistical power (1–*β*) of 85%, the required sample size was calculated to be 186 participants. To account for a potential 10% dropout rate, the total planned enrollment is set at 205 patients with chronic HF. However, the study team elected to enroll 300 patients to further strengthen the statistical robustness of the analysis. This larger planned sample provides several advantages:

1. it compensates for potential attrition, incomplete adherence, or missing data;
2. it increases the precision of effect estimates in a clinically heterogeneous HF population;
3. it enhances the stability of exploratory subgroup analyses across LVEF phenotypes (HFrEF, HFmrEF, HFpEF);
4. it improves the reliability and generalizability of machine-learning models derived from multimodal wearable data.

Thus, while 205 participants represent the minimum requirement based on power analysis, the planned enrollment of 300 patients ensures greater analytical robustness and supports the broader aims of the SMART-CARE project.

#### 2.6.4 Handling of missing data and dropouts

Missing data and incomplete follow-up will be handled according to established recommendations for observational cohort studies. For clinical, biochemical, and echocardiographic variables, the pattern and mechanism of missingness will be evaluated. When data are missing at random (MAR), multiple imputation using chained equations (MICE) will be applied to reduce bias and preserve statistical power. In case of non-random missingness, sensitivity analyses will be performed using complete-case analysis and worst-case/best-case scenarios. For time-to-event analyses, dropouts will be treated as censored observations at the last available follow-up. Device adherence will be quantified as the percentage of monitoring days with valid wearable data; predefined thresholds for minimum acceptable adherence will be applied in sensitivity analyses. Per-protocol and intention-to-monitor analyses will be conducted to assess the robustness of the findings with respect to missing data and incomplete device use.

## 3 Expected results

We anticipate that AI-based remote monitoring with the integration of wearable devices will lead to a ≥20% reduction in hospital admissions over six months compared to standard care. Furthermore, we expect improvements in secondary outcomes, including enhanced quality of life, reduced therapy-related adverse events, and more favourable trends in biochemical and echocardiographic parameters. By enabling early detection of clinical deterioration and supporting timely intervention, the SMART-CARE system is expected to offer a more proactive, individualized, and data-driven approach to managing CHF. These findings may support the integration of wearable technologies and AI into routine clinical practice for heart failure patients, potentially reducing the burden on healthcare systems and improving long-term patient outcomes.

## 4 Discussion

The SMART-CARE study addresses a critical unmet need in the management of CHF: the timely identification and intervention of subclinical deterioration. In fact, despite advances in pharmacologic and device-based therapies, CHF continues to be a leading cause of hospital admissions and mortality worldwide. Traditional follow-up models, based primarily on scheduled clinical visits, often fail to capture early warning signs, resulting in delayed therapeutic action and potentially avoidable decompensations. Recent developments in digital health have opened new frontiers in CHF care. Wearable sensors capable of continuous physiological monitoring, when integrated with AI systems, have the potential to revolutionize disease surveillance and promote truly personalized medicine. The SMART-CARE trial leverages these technologies to evaluate whether a real-time, AI-driven approach can improve clinical outcomes by facilitating earlier interventions and reducing hospitalization rates.

Our study uses a CE-certified, minimally invasive wrist-worn device (EmbracePlus) as the core technology for continuous multimodal monitoring, capable of collecting biometric data—including HRV, RR, electrodermal activity, and sleep patterns—in real time. In addition, other wearable devices (e.g., Movesense MD sensor, Polar H10 chest strap) and non-invasive multiparametric monitors are integrated into the SMART-CARE ecosystem to broaden the spectrum of physiological parameters captured. This multimodal strategy allows a more comprehensive assessment of patient status, enabling advanced AI algorithms to detect subtle trends indicative of early heart failure decompensation. When abnormalities are identified, the system generates alerts that prompt timely clinical assessment, either through teleconsultation or an in-person visit, thus supporting early intervention and optimized treatment management.

Beyond its clinical implications, SMART-CARE also aims to create a large, high-quality dataset integrating patient history, biochemical markers, imaging, and multimodal sensor-derived data from different wearable devices. The use of a heterogeneous set of technologies—including wrist-worn, chest-worn, and multiparametric non-invasive monitors—provides complementary physiological information, thereby strengthening the granularity and robustness of the collected dataset. This comprehensive data integration will serve as the foundation for developing predictive algorithms and composite risk scores (multimarker models) capable of forecasting disease trajectory and informing precision therapy in future studies.

The SMART-CARE design is expected to demonstrate several key strengths, including its real-world applicability, multicenter collaboration, and patient-centred outcome measures, such as quality of life and monitoring of adverse events. If successful, SMART-CARE could support the integration of wearable health technologies into routine CHF care pathways, facilitating a shift from reactive to preventive management. The findings may also inform healthcare policy regarding the scalability of telemonitoring platforms and resource allocation for chronic disease management. Finally, the modular integration of different wearable devices within the SMART-CARE platform underscores its scalability and adaptability, allowing the system to be tailored not only to the diverse phenotypes of heart failure but also to other chronic conditions where continuous remote monitoring may provide clinical benefit.

## 5 Limitations

This study has several potential limitations that should be acknowledged. First, the non-randomized design may introduce selection bias, although the use of statistical adjustment methods (including multivariable regression and propensity score analyses) is expected to mitigate this risk. Second, the adoption of wearable devices may be influenced by a digital divide, with older or less digitally literate patients potentially underrepresented in the intervention group, which could limit the generalizability of the findings. Third, the reliance on continuous biometric monitoring and AI-driven alert generation raises the possibility of false positives, which may result in unnecessary clinical evaluations and potentially increase healthcare utilization. Conversely, false negatives cannot be excluded, with the risk of delayed detection of clinical deterioration. Finally, adherence to wearable use may vary over time, and incomplete data capture could affect the robustness of the analyses.

## Data Availability

The original contributions presented in the study are included in the article/Supplementary Material, further inquiries can be directed to the corresponding author.
